# Pulsed Electromagnetic Field Therapy as a Complementary Alternative for Chronic Pelvic Pain Management in an Interstitial Cystitis/Bladder Pain Syndrome Patient

**DOI:** 10.1155/2019/5767568

**Published:** 2019-12-26

**Authors:** Tyler L. Overholt, Christina Ross, Robert J. Evans, Stephen J. Walker

**Affiliations:** ^1^Department of Urology, Wake Forest Baptist Health, USA; ^2^Wake Forest Institute for Regenerative Medicine, USA

## Abstract

Interstitial cystitis/bladder pain syndrome is a chronic pelvic pain condition with no known etiology that affects millions of women and men in the United States. Current management can be aggressive for individuals who are refractory to less invasive options, often resulting in the use of opioid narcotics and/or surgical procedures under general anesthesia, with higher risks and side effects to patients. Pulsed electromagnetic field therapy is a noninvasive therapeutic strategy that is thought to reduce inflammation and pain via alteration of cellular function and microcirculation. This therapy has demonstrated efficacy in management of other chronic pain syndromes including fibromyalgia and chronic low back pain. Herein, we describe a case of pulsed electromagnetic field therapy for management of interstitial cystitis/bladder pain syndrome that resulted in decreases in pelvic pain, burning with bladder filling, and other nonpelvic pain symptoms. This case provides support for a formal clinical trial to evaluate the efficacy of pulsed electromagnetic field therapy for the management of chronic pelvic pain in interstitial cystitis/bladder pain syndrome.

## 1. Introduction

Interstitial cystitis/bladder pain syndrome (IC/BPS) is a chronic pelvic pain condition that affects millions of women and men in the United States [1, 2]. This disease is vastly heterogeneous across the affected population, and there is no clearly defined etiology of the underlying disease pathophysiology. Given the widespread variety of disease presentation, finding an effective management strategy is often difficult. This commonly leads to the utilization of more invasive options including long-term opioid narcotic use and aggressive surgical procedures under general anesthesia, resulting in billions of dollars in healthcare costs for management of these patients' symptoms [2, 3]. A minimally invasive therapeutic option for IC/BPS and related symptoms is critically needed for this patient population. Herein, we describe a case of the use of pulsed electromagnetic field (PEMF) therapy for management of chronic pelvic pain in an IC/BPS patient.

## 2. Case Presentation

This is a 29-year-old female who was first diagnosed with IC/BPS in 2011. Her past medical history includes chronic pelvic pain for more than ten years, dyspareunia, migraine headaches, depression, anxiety, panic attacks, and asthma. Since her diagnosis, management across the spectrum of the American Urological Association (AUA) guidelines for IC/BPS has been attempted, spanning from minimally invasive to invasive strategies. These include pelvic floor physical therapy, topical lidocaine injections, several oral pharmacological therapies such as opioid narcotics, and surgical procedures such as cystoscopic hydrodistention. Almost all AUA recommendations have been tried in this patient with limited success. In addition, she has undergone detailed gynecological and gastrointestinal evaluations including an exploratory laparotomy with little success in finding additional causes for her pelvic pain in addition to IC/BPS and/or relief for her symptoms.

**Figure 1:**
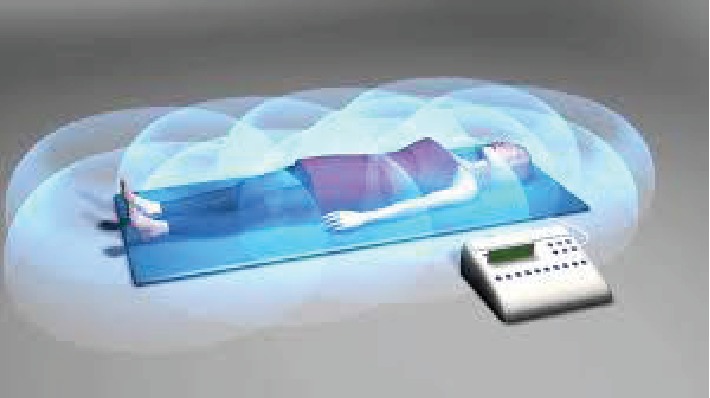
A pulsed electromagnetic field device. PEMF devices consist of a mat designed to target the entire body for generalized exposure, as well as a belt that can be specifically laid over the pelvis to provide targeted therapy to specific areas.

During the summer of 2018, she was first introduced, through a medical professional outside of our institution's health network, to a pulsed electromagnetic field (PEMF) device as an alternative therapeutic strategy for pain management. PEMF is administered via an FDA-registered device that is thought to function by affecting cellular interactions and the microvascular circulation of targeted organs to reduce overall inflammation and pain. The PEMF device consisted of a mat designed to target the entire body, as well as a belt that was laid specifically over the pelvis to provide generalized exposure as well as targeted therapy (Figure 1). This device was programmed as per standard device protocols to a frequency of 33 Hz, a sinusoidal waveform, and to an intensity setting ranging from 3.5 to 35 microtesla. Each treatment session was eight minutes total in duration [4, 5].

For several months, the patient reported using this device at least one or two times weekly. She specifically described a two-week period where she used this device every morning and evening and reported a significant, continuous relief of many of her pelvic pain-related symptoms. Most notably, she reported experiencing reductions in bloating, burning with bladder filling, and her overall pelvic pain. She additionally reported improvements in her energy level and her ability to participate in regular physical exercise as well as a reduction in migraine headache episodes. Symptoms that were not changed with the use of this device were urgency and frequency of urination.

During the entire duration of use, the patient reported no apparent side effects to this therapy and found it to be a minimally invasive and easy to use therapeutic strategy for symptom relief. Due to the inability of the patient to purchase her own device, given the very expensive out-of-pocket price, the patient was not able to continue use for this reason alone. Following discontinuation of use, the patient reported a return of symptoms to her baseline level within 12-24 hours.

## 3. Discussion

Interstitial cystitis/bladder pain syndrome (IC/BPS) is a chronic pelvic pain disorder that is thought to affect 3-8 million women and ~4 million men in the United States [1, 2]. While this disease is vastly heterogeneous in presentation, the most common clinical presentations include lower urinary tract symptoms such as urgency, frequency, and dysuria, as well as bladder/pelvic pain most notably with bladder filling [1, 2]. Additionally, many IC/BPS patients experience nonbladder symptoms that are closely related to their pelvic symptoms including fibromyalgia, irritable bowel syndrome, endometriosis, and mental health disorders [1, 2].

There are several hypotheses regarding the etiology of IC/BPS. One theory with a large and growing body of research involves the structure and function of the urothelial cell layer of the bladder [6]. Studies have shown that urothelial cells from IC/BPS patients proliferate slower and enable an increased amount of leaking across cell-cell tight junctions when compared to non-IC/BPS controls [6]. Other theories involve detrusor mastocytosis with infiltration of inflammatory cells and subsequent pain [7], nerve fiber overproliferation and neurogenic inflammation [8], and central nervous system (CNS) involvement through central sensitization and amplification of the pain response [9]. While there are many different hypotheses, it is thought that multiple pathophysiologic mechanisms may be occurring simultaneously, indicating a likely multicomplex etiology of IC/BPS in affected patients.

Given the unclear underlying pathophysiology, management strategies are predominantly centered around pain control and symptom reduction [1, 2]. The American Urological Association (AUA) most recently amended guidelines for IC/BPS management in 2014 that present potential treatment strategies as first through sixth line options [2]. Minimally invasive strategies such as pelvic floor physical therapy and stress/behavior modifications have the highest grade evidence for efficacy, but many patients require more aggressive management strategies [2]. These include multimodal, oral pharmacological therapy including opioid narcotics and surgical therapies under general anesthesia such as cystoscopic hydrodistention and cystectomy [2]. Even for the guidelines considered first line, the data supporting these strategies are mostly based on observational, retrospective studies, and demonstration of efficacy is often weak [2].

Pulsed electromagnetic field (PEMF) therapy, based on Faraday's Law of electromagnetism, is a combination of frequency, intensity, and time duration used to alter cellular function and restore the cells to normal rhythms [10]. Modulation of cellular function via the electromagnetic field has been reported to improve overall cell-cell interactions and microvascular circulation of targeted organs to improve inflammation and pain [10]. PEMF devices have been clinically studied in other patient subgroups with successful demonstration of symptom resolution. For example, randomized, controlled trials have showed that PEMF device therapy resulted in improved mobility, pain scores, and energy level in fibromyalgia and chronic musculoskeletal pain patients [11, 12]. Another randomized, controlled trial has shown that PEMF therapy resulted in significantly improved scores on validated pain assessment questionnaires and a reduction in disability in patients with chronic low back pain [13].

Pelvic pain reduction in a difficult patient through the use of PEMF therapy demonstrates clinical usefulness and argues for further evaluation of the effects of PEMF therapy on chronic pelvic pain via clinical trials. A limitation of this report regarding the efficacy of PEMF therapy in this patient is a lack of a standardized approach to quantify symptom improvement. Further evaluation via a controlled trial is needed to assess symptom relief through standardized measures as compared to a control group. This would include validated instruments for quantifying symptom reduction/resolution such as IC/BPS questionnaires and depression and anxiety questionnaires. Additionally, a controlled trial would be necessary for assessment of optimal dosing and duration of treatment parameters. In summary, PEMF therapy is a minimally invasive therapeutic strategy for treating chronic pain, with no reported side effects, that provided benefit to an IC/BPS patient who experienced chronic pelvic pain refractory to almost all standard therapeutic strategies for pelvic pain relief.
